# The Influence of Cholesterol on Membrane Targeted Bioactive Peptides: Modulating Peptide Activity Through Changes in Bilayer Biophysical Properties

**DOI:** 10.3390/membranes14100220

**Published:** 2024-10-17

**Authors:** Juan M. Giraldo-Lorza, Chad Leidy, Marcela Manrique-Moreno

**Affiliations:** 1 Chemistry Institute, Faculty of Exact and Natural Sciences, University of Antioquia, A.A. 1226, Medellin 050010, Colombia; juan.giraldo77@udea.edu.co; 2 Biophysics Group, Physics Department, Universidad de los Andes, Bogotá 111711, Colombia; cleidy@uniandes.edu.co

**Keywords:** cholesterol, bioactive peptides, membrane fluidity, cholesterol–peptide interactions

## Abstract

Cholesterol is a biological molecule that is essential for cellular life. It has unique features in terms of molecular structure and function, and plays an important role in determining the structure and properties of cell membranes. One of the most recognized functions of cholesterol is its ability to increase the level of lipid packing and rigidity of biological membranes while maintaining high levels of lateral mobility of the bulk lipids, which is necessary to sustain biochemical signaling events. There is increased interest in designing bioactive peptides that can act as effective antimicrobial agents without causing harm to human cells. For this reason, it becomes relevant to understand how cholesterol can affect the interaction between bioactive peptides and lipid membranes, in particular by modulating the peptides’ ability to penetrate and disrupt the membranes through these changes in membrane rigidity. Here we discuss cholesterol and its role in modulating lipid bilayer properties and discuss recent evidence showing how cholesterol modulates bioactive peptides to different degrees.

## 1. Introduction

Eukaryotic lipid membranes are structurally intricate, possessing hundreds of lipid species with a wide range of physicochemical properties [1]. They have a remarkable asymmetric lipid distribution, variations in acyl chain lengths and saturation levels, and variations in headgroup composition and geometries. The different chemical structures found in these lipid molecules influence several membrane properties, such as lateral mobility, mechanical strength, intrinsic curvature levels, and lateral organization, characterized by the presence of rafts or clusters [1]. Lipids that make up eukaryotic membranes can be divided into three categories according to their structure and function: glycerophospholipids, sphingolipids, and sterols. Glycerophospholipids are characterized by a glycerol backbone, with two acyl chains attached through ester bonds, and a headgroup characterized by the presence of a phosphate group that is negatively charged at physiological pH. Headgroup composition varies, leading to different net charges, sizes, and polarities, and phosphatidylcholine and phosphatidylethanolamine are found to be the most abundant headgroup species in eukaryotic membranes [2]. Sphingolipids share structural similarities with glycerophospholipids. However, the chemical structure does not contain a glycerol backbone. Instead, a sphingosine attaches a fatty acid through the amide group in the sphingosine, while the headgroup is attached to the hydroxyl of the sphingosine. Sphingomyelin contains a phosphate group and a choline in its headgroup, making it very similar to phosphatidylcholine. Sphingomyelin is found to be highly enriched, mainly in the extra-cellular leaflet of the plasma membrane, where it plays a role in the formation of lipid rafts [3].

Among sterols, cholesterol is the main sterol found in eukaryotic membranes, where it makes up 10–50% of total lipids in eukaryotic cells and has a range of effects on overall membrane properties [4]. It has long been noticed that the biophysical and biochemical properties of the membrane are diversely affected by the lipid composition and organization [5]. However, the unique structure of CHO with a polar head and rigid ring structure in its hydrophobic tail renders the molecule particularly important in regulating biophysical properties that affect lipid packing, fluidity, lateral organization, and the shape of phospholipid bilayers [6]. As a regulator of membrane physical properties, it is a crucial component of many cellular functions, including vesicular transport and the signal transduction pathways [7]. CHO plays important roles in the organism as the precursor of several hormones, bile acids, and vitamin D, holds the membrane proteins in a functional conformation, and is also an important mediator in cell signaling pathways [8]. Some studies also show that cholesterol takes part in the communication between intra- and extra-cellular processes as a modulator of various processes [9].

The rigid ring structure found in cholesterol induces increased lipid packing by forcing the alignment of the lipid acyl chains of neighboring phospholipids, leading to ordering of the acyl chain conformations. The hydroxyl group in the molecule positions the molecule close the hydrophilic/hydrophobic interface of the bilayer, with the ring structure extending into the acyl chain region and positioning itself parallel to the acyl chains, resulting in a reduction in rotational conformations of the chains. Acyl chain alignment induces an increase in bilayer thickness in regions enriched in cholesterol. These structural changes lead to a modulation of the mechanical properties of the membrane reflected in an increase in the bending rigidity of the membrane. However, since cholesterol acts as a spacer that separates neighboring phospholipids, it is also able to maintain lateral mobility of phospholipids and proteins in the membrane even while inducing an increase in membrane rigidity. For this reason, cholesterol has the remarkable property of increasing the mechanical strength of the lipid bilayer without strongly affecting the lateral mobility of lipid and protein components, which is crucial for maintaining essential processes, such as signaling dynamics. An additional remarkable trait of this molecule is that cholesterol can induce phase separation in the lipid membrane, which results in the formation of lipid microdomains, also known as membrane rafts, that are used by a variety of cells to segregate proteins in order to organize signaling events and endocytosis.

With regards to the modulation of mechanical properties, a prime example of the importance of cholesterol is seen in red blood cells (RBCs). RBCs contain very high cholesterol levels, close to 50 mol%, which is near the saturation concentration point of cholesterol in the membrane. This cholesterol concentration is too high to maintain phase separation, in which case the red blood-cell plasma membrane is found in a one liquid-ordered phase enriched in cholesterol. For this reason, the main purpose of these high levels appears to be an increase in the mechanical strength of the RBC membrane to prevent damage during transport in the circulatory system.

Cholesterol, the only sterol present, is found in eukaryotic membranes, where it makes up to 10–50% of total lipids and has a range of effects on overall membrane properties [4]. A molecule capable of modulating the physicochemical properties of the membrane has the potential to modulate the activity of membrane-active molecules, like bioactive peptides (BAPs). These molecules are fundamental components of the immune system of all living organisms [10]. They are small amino acid sequences with multiple effects on body function [11,12,13,14,15,16]. These physiological properties have led to BAPs being considered a promising pharmaceutical tool against several diseases [17,18,19,20,21,22,23,24,25,26]. It has been widely accepted that most BAPs exert their activity by interacting with the phospholipids of the membranes, destabilizing the membrane of the target cells. This mechanism is difficult for the target cells to counteract, and is responsible for the rapid effect of the peptides [10].

Many membranes targeted bioactive peptides are characterized by binding and inserting into biological membranes, either to traverse the membrane and enter the cytoplasm to reach intracellular targets, or to group in an inserted state to induce pore formation and leakage in the membrane. The insertion process always requires a local mechanical deformation of the membrane to take effect. Therefore, the lipid composition of target membranes, which affects the bending modulus of the lipid bilayer, is a key factor in determining the efficacy of bioactive peptides [27,28]. A regulator of membrane rigidity, cholesterol is a major structural component of the membrane, which can modulate the interactions between BAPs and lipid membranes by affecting the peptides’ ability to penetrate and disrupt the membranes. In the case of antimicrobial peptides, the potential inhibition of BAPs by cholesterol will have direct relevance for how BAPs interact differentially with the host cells that do synthesize cholesterol, compared to bacterial cells that do not. This leads to intriguing questions about the potential roles of cholesterol–peptide associations as a way to protect the cells producing the peptides in order to fight pathogens. This review will focus on the general characteristics of BAPs, the cell membrane structure, and the effect of cholesterol and the consequences of its interaction with peptides. This information can be useful in the design of BAPs by helping the peptide design process to take into account information related to the role of cholesterol in the modulation of BAP activity.

## 2. Bioactive Peptides (BAPs)

BAPs are a heterogeneous group of molecules, expressed in organisms ranging from bacteria to humans, that provide an adaptive advantage for the innate host defense system of these organisms by exerting countless biological effects through several mechanisms [29]. BAPs are mainly known for their direct interactions with and effects on microbial membranes, but many peptides can also target an array of other key microbial components, such as lipopolysaccharides [30], teichoic acids, peptidoglycans [31], nucleic acids [32], and proteins [33], due to their exceedingly high affinity and absence of rigid binding specificity [34,35].

### 2.1. Physicochemical Properties of BAPs

Due to the combinatorial arrangement of amino acids within a particular primary sequence, there is an exponential number of potential primary structures. The amino acids which conform a sequence can be neutral, charged, polar, hydrophobic, and even synthetically modified. The structure–activity analysis of numerous BAPs has revealed that their biological activities are linked to a number of common features. Specifically, they are short sequences of 5–50 amino acid residues (less than 10 KDa), mainly cationic, amphipathic, and with approximately 50% of residues in the sequence being hydrophobic [36]. Despite these common characteristics, they are highly diverse with respect to their activity against bacteria [37], viruses [38], parasites [39,40,41], cancer [42], fungi [43,44], etc. Most BAPs possess a net positive charge at physiological pH because of their high content of basic residues, such as arginine and lysine. The charge of the peptides, located on the side chains of the residues, enables multiple interactions, such as hydrogen bonding, salt bridges and dipole interactions with the anionic charged groups of the microorganism membranes, a step that has been accepted [45,46] as necessary for them to exert their activity [47,48]. Most of the biologically active sequences reported in the databases possess a net positive charge, ranging from +3 to +9 [45,46].

The hydrophobicity and amphipathicity of bioactive peptides are characteristics of considerable interest. Extensive study has found that peptides with higher hydrophobicity penetrate the hydrophobic core of cell membranes, leading to cell disruption through the necrotic mechanism [49,50]. Several studies have attempted to replace hydrophobic and neutral amino acids with positively charged residues, such as lysine, leucine or phenylalanine, on the polar and non-polar faces of α-helical peptides [51]. Consequently, cationic peptides with moderate hydrophobicity have shown increased cytotoxicity against target cells [46,52]. Amphipathicity is defined as the distribution of polar and nonpolar residues across the backbone of the polypeptide chain. Essentially, an amphipathic peptide has hydrophilic and hydrophobic regions, resulting in water-repelling (nonpolar) and water-attracting (polar) regions. The structural arrangement of these regions allows BAPs to interact with and disrupt the target membranes, which is crucial for their biological activity. Adverse interactions with eukaryotic membrane counterparts then depend upon a delicate balance between hydrophobicity and charge. Amphipathicity, together with net charge and length, determines the disposition of the peptide towards the cell membrane and therefore the mechanism by which the peptide exerts its biological activity. Multiple BAPs, with overlapping but non-identical sequences, activities and mechanisms of action, can often act cooperatively to facilitate the killing of invading pathogens [53].

### 2.2. Structural Characteristics

**Figure 1:**
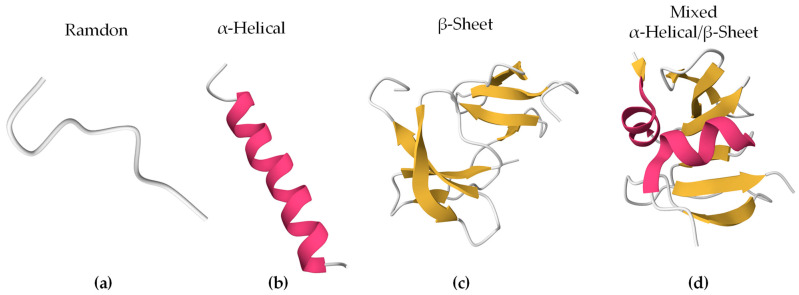
Secondary structures of representative BAPs using PyMOL 3.0. In brackets are the PDB ID and the origin of the peptides. The colors represent the secondary structures: (**a**) Indolicidin (PDB ID 8IS3, *Bos taurus*), (**b**) Magainin 2 (PDB ID 2MAG, *Xenopus laevis*), (**c**) Human β-Defensin-4 (PDB ID 5KI9, *Homo sapiens*), and (**d**) Human β-Defensin-2 (PDB ID 1FD4, *Homo sapiens*).

Bioactive peptides can adopt different secondary structures (Figure 1). Some peptides are unstructured upon interaction (random/coil), while others adopt a more stable structure before or after they associate with lipids. The largest population of reported secondary structures of BAPs is α-helix, followed by random coil and β-sheet [54]. These elements often coexist in the same peptide.

## 3. Eukaryotic Membranes

### 3.1. Structure of Membranes

**Figure 2:**
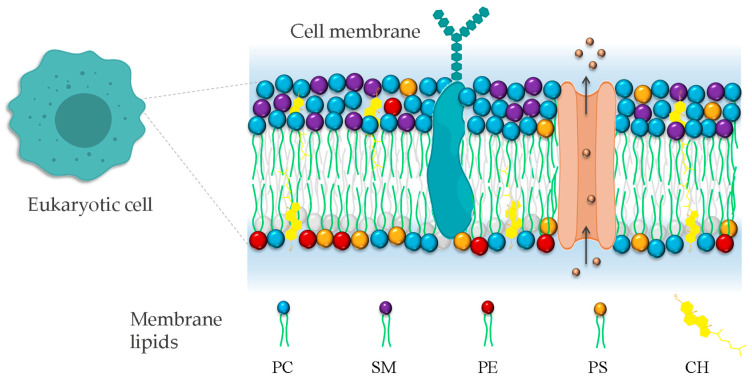
Representation of eukaryotic cell plasma membrane. The phospholipid bilayer contains all molecules, including phospholipids, proteins, and cholesterol. Phosphatidylcholine (PC), phosphatidylethanolamine (PE), phosphatidylserine (PS), and sphingomyelin (SM). The irregular representation of the lipid acyl chains of lipids denotes the fluid nature of the bilayer.

In addition to populating many unicellular communities, eukaryotic cells are the building blocks of complex multicellular living systems. Eukaryotic cells require a demarcating structure that separates them from their external environment and envelopes their internal membrane-bound organelles. This structure is called plasma membrane, and is consistent in its construction across the *Eukarya* [55]. The classic model of bio-membranes was proposed by Singer and Nicolson, who named it the fluid mosaic model [56]. In this model, the membrane was described as an unstructured liquid–crystal characterized by a disorderly and chaotic arrangement of molecules in a two-dimensional liquid–crystal matrix. However, with advances in membrane biophysics, the model was complemented and improved, extending the description and the understanding of the membrane organization by including the notion of a heterogenous arrangement of lipids and proteins on the membrane surface due to the formation of lipid domains. The cell membrane is currently described as a heterogeneous two-dimensional semi-fluid barrier that acts as a matrix containing a population of lipids, proteins, and glycolipids with transitory mobility (Figure 2) [57].

The main structure of the membrane consists of lipids arranged in a bilayer. These lipids are non-covalently associated in the lipid bilayer. Their interactions are weak entropic and transient dipole in nature, which leads to constant lateral motion, characteristic of a liquid state. In addition, there are other molecules, such as proteins, on the membrane (peripheral) and within it (integral), that are associated with the lipid bilayer by non-covalent interactions and therefore differing degrees of mobility. These proteins have functions as diverse as those of pore formers, signal transducers, and enzymes. Thus, the membrane is more than just two-dimensional and is a complex array of dynamic patterns involving inter- and intra-molecular interactions. The integration of proteins and glycolipids into the cell membrane reflects one of the priorities of the signal hypothesis, that the membrane permits the controlled transmission of biological information in either a secreted, (open) or transduced (closed) channel manner [58].

### 3.2. Lipid Composition

**Figure 3:**
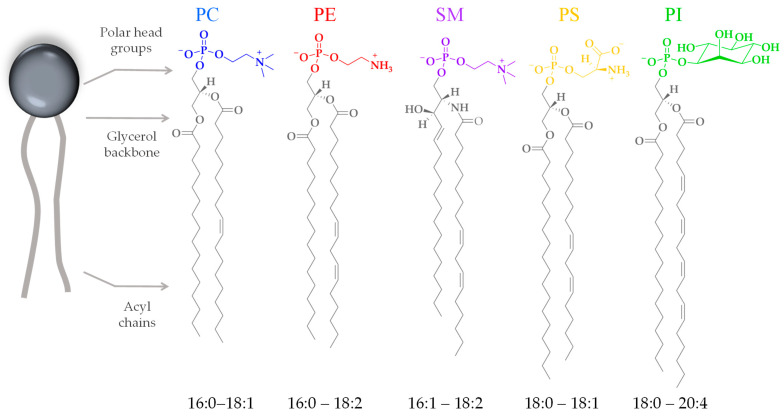
Chemical representation of the head groups and acyl chains of the most abundant phospholipids of eukaryotic cell membranes. Phosphatidylcholine (PC), phosphatidylethanolamine (PE), phosphatidylserine (PS), sphingomyelin (SM), and phosphatidylinositol (PI). The acyl chains range from fully saturated to multiple unsaturated.

Cell membranes are composed mostly of proteins and lipids. There are several families of lipids present in the eukaryotic plasma membrane, showing varying compositions depending on the cell type. In human cells, the main families are phospholipids and glycolipids. Phospholipids are composed of a negatively charged phosphate group, a complex alcohol group (glycerol moiety), and two fatty acids. Phospholipids are described as amphipathic molecules because of their double-nature structure: the polar head group is hydrophilic, and the non-polar tails are hydrophobic. The properties of membrane lipids, such as their charge distribution, molecular size, and molecule-to-molecule interactions, depend on their head group and tail chemistry. The phosphate-containing portions of the molecule are widely diverse (Figure 3). This moiety can be charged or polar, and so it interacts attractively with the surrounding water through polar interactions. The long, hydrophobic fatty acid tails, on the other hand, do not present a permanent charge polarity, which impedes the fatty acids from interacting with the surrounding water through polar interactions. The acyl chains can also be diverse in terms of their length and the presence of unsaturated bonds (Figure 3), influencing the level of lipid packing. After the lipids assemble into a bilayer, it will feature a hydrophilic outer surface on both leaflets, facing the water environment, and a hydrophobic inner core characterized by a fluid hydrocarbon environment at physiological temperatures.

The plasma membranes and organelle membranes of the different cell types found in the human body exhibit widely diverse lipid compositions. Lipid bilayers have many functions, and can have specific features depending on the functional characteristics that the different membranes have to fulfill [1]. Corresponding to these functions, different lipid types are present in the different cavities and leaflets of each membrane. Lipids, such as sphingomyelin and cholesterol, found in the outer leaflet of the plasma membrane of T-cells, help organize transmembrane proteins in charge of detecting and eradicating pathogens [59], while cardiolipin is found in the inner membrane of mitochondria, providing the spontaneous curvature needed to induce the characteristic folding of this membrane [60].

**Figure 4:**
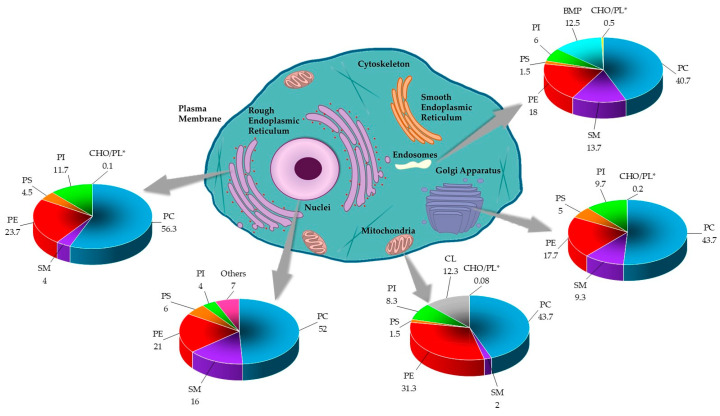
The lipid composition of different organelles throughout the eukaryotic cell. The lipid data in the graphs are presented as a percentage of total phospholipids (PL) in mammals. The cholesterol content is presented as the molar ratio of cholesterol (CHO) with respect to the PL [2,3,61]. * Data was reported in CHO/PL ratio.

Lipids are important integral components of the organelles of eukaryotic cells. They are considered to determine the function of these subcellular structures [3]. The endoplasmic reticulum is equipped with a characteristic lipid metabolism with de novo synthesis of phospholipids. The metabolism of de novo phosphatidylethanolamine and phosphatidylcholine, the most synthesized phospholipids of this compartment, also involves their acylation. The secretory pathway next to the endoplasmic reticulum ends with the Golgi apparatus, which has a different lipid metabolism from the precursor [2]. In particular, the metabolism of sphingomyelin, which increases in the Golgi apparatus, and that of the phospholipid characteristic of this compartment, is remarkable. The process of intra-Golgi membrane trafficking, and other processes that Golgi is not associated with, would modify the lipid composition of this compartment, whose function would not only provide lipids and proteins for the plasma membrane but also distribute certain lipids, including sphingolipids, in a classic way toward the apical layer, coupled with the formation of a basal loop of the trans-Golgi [3]. Figure 4 summarizes the lipid composition reported for the eukaryotic cell organelles.

**Figure 5:**
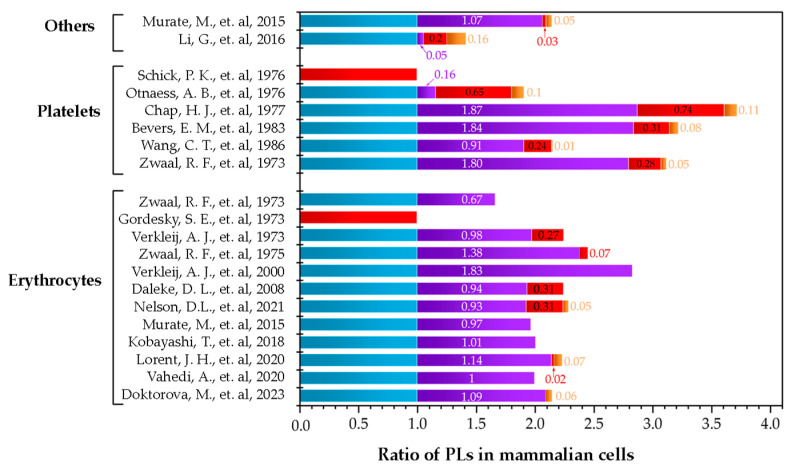
Summary of publications that quantified the phospholipid distribution in mammalian cells. Others are fibroblasts and cancer cells. The colors correspond to PC (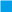), SM (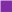), PE (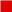), and PS (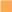). Data are presented in ratios based on PC abundance.

The complexity of cell membranes suggest that the lipid models used in biophysical studies must be prepared as multi-component lipid systems based on the composition of the membrane under study. This would lead to more representative models, which can reflect more biologically relevant conditions [62,63,64]. Nevertheless, when deciding what proportions of lipids to use as representative of plasma membranes, the reported distribution of phospholipids is very broad. To visualize the different possibilities that are available, Figure 5 summarizes different works that reported the distributions of lipids in various cell types. Additionally, in Table 1, the original data are presented, which includes how the authors reported the using different techniques for the quantification and different methods for the extraction of the lipids of the outer layer of the membrane. Regarding the amount of cholesterol, the published data are quite imprecise. Most publications do not include the quantification of sterol leading to gaps in the information needed to determine the proportions to include in a potential artificial lipid model. As cholesterol is such an important molecule in the regulation of biophysical properties of the membrane, a clearer understanding of cholesterol content for the different biological membranes is necessary.

## 4. Cholesterol

**Figure 6:**
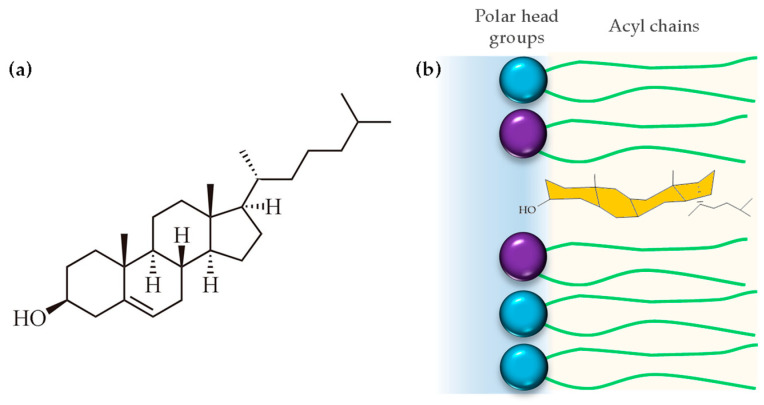
(**a**) Chemical structure of cholesterol and (**b**) disposition of cholesterol into the hydrophobic region of the cell membrane.

Cholesterol is a 27-carbon compound with a unique structure containing a central sterol core made of four hydrocarbon rings, a hydroxyl group, and a hydrocarbon tail (Figure 6a). The center sterol nucleus or ring is a feature of all steroid hormones. The cholesterol molecule is composed of three distinct regions: a small polar hydroxyl group, a rigid, plate-like steroid ring, and a flexible iso-octyl chain. The iso-octyl chain, which has a much smaller cross-sectional area than the rigid ring, is located at the lipid core of the membrane, and presents conformational freedom, similar to the lipid acyl chains (Figure 6b). When cholesterol incorporates into the membrane, it is expected that the polar hydroxyl group will be positioned between the polar headgroups of phospholipids, while the rigid ring structure extends into the hydrophobic acyl chains, aligning with the carbon atoms [84]. However, biophysical studies using polyunsaturated phosphatydilcholines (20:4–20:4) and neutron diffraction experiments showed that cholesterol can even lie in the membrane midplane. Harroun et. al. attributed the change in location to the high level of disorder of polyunsaturated fatty acids, which is incompatible with proximity to the rigid steroid moiety in its usual upright orientation [85].

### 4.1. Cholesterol as a Physiological and Structural Component

Cholesterol, an essential component of eukaryotes, is a precursor for several steroid hormones, vitamin D3, and bile acids [8]. This neutral lipid is well known for being implicated in several medical issues, particularly heart diseases [86]. However, it also mediates several physiological functions, particularly in animal cells. After absorbing cholesterol from a healthy diet, the body ceases to synthesize cholesterol de novo, reducing endogenous cholesterol production and balancing the variation [87].

Organisms use cholesterol for the maintenance of cell membrane stability [88], increasing mechanical resistance [89], regulating membrane internalization [90,91], membrane trafficking following exocytosis [92], and modulating the formation of endocytic synapsis [93,94]. The cholesterol molecule plays a crucial role in the spatial arrangement of the lipid bilayer. García-Arribas reported that the addition of cholesterol to the membrane imparts stability and rigidity, preserving membrane architecture [95]. However, in addition to this mechanical function, cholesterol modulates the functional organization of the membrane by inducing the formation of mesoscopic domain regions on the plasma membrane, known as lipid rafts, that serve to segregate relevant transmembrane and peripheral proteins into functional regions [7]. These domains are formed due to thermodynamic phase separation of lipid components in the plane of the membrane.

Cholesterol, sphingomyelin, and glycosphingolipids segregate to form liquid-ordered phase regions segregated from liquid-disordered phase regions. The liquid-ordered phase receives its name from the condensed ordering of the acyl chains, which is induced by the presence of specific lipids interacting with cholesterol, which otherwise maintains a mobile liquid state of the lipids in the plane of the membrane. The liquid-disordered phase maintains a high level of conformational disorder of the acyl chains in addition to behaving as a liquid in the plane of the membrane. For this reason, this domain structure is denominated as liquid–liquid coexistence. This segregation of these phases in the plane of the membrane plays several crucial physiological roles in a diverse range of cellular processes [96]. The main structural feature that promotes this segregation is the preferential interaction of cholesterol with sphingolipids. Cholesterol occupies spaces between the acyl chains and polar headgroups in the lipid bilayer, as well as influencing lipid interdigitation between different lipid leaflets [95]. Since cholesterol is rigid and planar, cholesterol will prefer to accommodate itself next to the extended acyl chains of sphingomyelin, which lack cis-double bonds. The cis-double bonds normally present in phosphatidylcholine lead to kinks in the acyl chains that result in a steric hindrance to the cholesterol lining up with these chains [97]. This preferred interaction with sphingomyelin leads to a straightening of the sphingomyelin acyl chains, which induces a local increase in bilayer thickness. The difference in bilayer thickness between the SM-rich regions and the PC-rich regions induces line tensions that lead to domain formation. Cholesterol is also an important mediator in cell signaling pathways. Some evidence also shows that cholesterol takes part in the communication between intra- and extra-cellular processes as a modulator of various processes. The nature of cholesterol presents a contradiction, and its dual functions have been studied extensively [98].

### 4.2. Cholesterol as a Membrane Fluidity Regulator

Cholesterol is a unique lipid, and an essential constituent of eukaryotic membranes. Although the role of cholesterol in biological systems has been studied for more than 100 years, the complete biophysical role of this lipid is not yet fully understood. One well-established role of cholesterol in biological systems is as a regulator of membrane biophysical properties [6]. It is well known that cholesterol down-regulates overall membrane fluidity, both through the formation of tighter packing inside membranes induced by the straightening of lipid acyl chains and by directly modulating the anomalous and chain-conformational lengths. This modulation is due to its large (3-hydroxyl) group and O linkage at the C3 position, which both set constraints that restrict local chain bending and improve steric packing within the bilayer [6]. At the same time, it also softens the anomalous chain tilted permutations, due to its shorter structure. Furthermore, the large tetracyclic A-ring of cholesterol enables it to pack tightly and with a high tilt angle against the normal membrane, with its ribose ester side-chain also acting to help further pack and deform the opposing leaflet. The more local scale effect of cholesterol-induced liquid-ordered domains is its imposition of a higher local curvature that attracts charged atoms (such as the phosphate in the head-group of PC and SM, and sterols) towards the center of the interfaces. The main effect of cholesterol, therefore, is liquid phase region stiffening, increasing the measurable two-dimensional elastic properties that encapsulate both monolayer stretching and compression properties, and vesicle surface bending and glycerol angular propulsion (stretch-coupling) [99].

Cholesterol is present at different concentrations in membranes, ranging from 10 to 50 mol%. The concentration of cholesterol affects the membrane properties. Low cholesterol concentrations have been detected in the nuclear envelope, membranes of rough and smooth endoplasmic reticulum, and membranes of Golgi apparatus [4]. These membranes are highly fluid and exhibit a moderate hydrophobic barrier, which allows the intracellular transport of substances and products. At high concentrations, cholesterol causes a decrease in membrane fluidity. For typical plasma membranes containing 10 to 30% cholesterol, the presence of cholesterol induces domain formation, as discussed above [100]. The presence of cholesterol reduces the tilt mobility of the lipid acyl chains and decreases the lateral diffusion of lipids in the bilayer [4,6].

Membranes with a high cholesterol content from 30 to 50 mol%, such as erythrocytes, are characterized by the hydrophobicity of the superior membrane, which increases its rigidity by minimizing trans-gauche isomerization (promoting an extended conformation) and reducing translational and rotational motion within the membrane. These properties are consistent with those expected for erythrocytes. Red blood cells must pass through narrow capillaries without losing molecules contained in the cytosol; its membranes must be able to avoid permeation of small molecules [101]. Extensive study has found that cholesterol limits oxygen diffusion across membranes, which helps regulate its proper transport in the circulatory system [102,103,104]. High concentrations of cholesterol simultaneously heighten hydrophobic barriers for polar molecules and strengthen rigidity barriers for nonpolar molecules [4]. Thus, the above observations suggest that cholesterol increases the thickness of the hydrophobic region of the bilayer, with the hydroxyl group of cholesterol located on the outer edge of the bilayer [6]. On the other hand, at low concentrations, cholesterol functions to increase the fluidity of membranes by preventing hydrophobic collapse of the acyl tails [105].

### 4.3. Cholesterol Asymmetry

Eukaryotic membranes are known for their asymmetric distribution of lipids, with distinct compositions in their bilayer leaflets. This asymmetric distribution generates an electrostatic potential that influences protein–lipid interactions, highlighting the significance of lipid asymmetry in the plasma membranes [106]. The active maintenance of lipid asymmetry through ATP-dependent transporters underscores its importance for cellular function, as evidenced by its association with a congenital bleeding disorder and its role in cell apoptosis, where the external exposure of the cytoplasmic leaflet lipid, phosphatidylserine, is key to this pathology [107].

One of the most recent works in the field showed, using quantitative lipidomics, that the phospholipid imbalance in red blood cells is facilitated by an uneven distribution of cholesterol between the leaflets, which quickly redistributes to alleviate leaflet stress. The asymmetric abundance and composition of phospholipids work together to concentrate cholesterol in the exoplasmic leaflet of the erythrocyte plasma membrane [5]. Blumer et al. demonstrated, through atomistic and coarse-grained simulations, that the number of lipids in each leaflet of the bilayer can be adjusted to minimize leaflet stress, highlighting the coupling between the bilayer’s two leaflets. Moreover, their findings suggest that cholesterol can reduce membrane asymmetry by flipping between leaflets and can influence certain properties such as the lipid packing parameter [108]. This insight underscores the intricate interplay between cholesterol and membrane asymmetry, shedding light on the complex nature of lipid distributions within the bilayer.

Additionally, different researchers have emphasized the significant influence of cholesterol on membrane proteins, particularly the functional effects of cholesterol on the superfamily of pentameric ligand-gated ion channels (pLGICs) [109,110]. Studies have demonstrated that cholesterol affects the ligand-recognition, gating, and ion permeation properties of hormone and neurotransmitter receptors, as well as ligand- and voltage-gated ion channels. Additionally, the presence of cholesterol in specific membrane domains dynamically compartmentalizes proteins, influencing cell-surface organization and protein trafficking [111]. Moreover, simulations have demonstrated the impact of bilayer asymmetry on bilayer properties, the influence of lipid components, and the adaptability of lipids to different environments [108].

The establishment of cholesterol asymmetry in eukaryotic membranes involves the action of flippases and floppases, which contribute to the uneven distribution of cholesterol across the membrane [112]. Studies have shown that the bilayer responds to asymmetry based on the composition and chemical structure of its lipids, with the number of lipids in each leaflet being adjusted to minimize leaflet surface tension. Additionally, cholesterol has been found to impact membrane asymmetry by reducing it for certain properties, such as overall membrane density and area per lipid, while increasing effects on others, such as lipid packing propensity [108,113]. Furthermore, the influence of cholesterol flip-flop on membrane properties, particularly the interleaflet coupling of cholesterol-enriched domains, has been investigated [114]. It was found that suppressing interleaflet cholesterol population significantly reduces the cholesterol density correlation between the leaflets of an average mammalian plasma membrane, suggesting an amplifying role of cholesterol in signal transduction across the leaflets [114]. These findings underscore the intricate mechanisms through which cholesterol asymmetry is established and its implications for membrane properties and functions.

## 5. Interaction of Cholesterol and BAPs

As explained above, cholesterol is an important modulator of the physicochemical properties of the lipid bilayer. One of the main effects that cholesterol induces in membranes is a change in their bending rigidity induced by the straightening of the lipid acyl chains, which results in an increase in membrane thickness, which is coupled to an increase in the bulk compressibility modulus of the membrane. Both of these properties, which affect the bending rigidity, are increased in the presence of cholesterol. Therefore, overall, membranes that contain cholesterol will have a higher free energy cost to bend. Since the insertion process of BAPs normally requires a high level of local deformation in the membrane, these changes in the mechanical properties of the membrane in the presence of cholesterol imply that there is a potential relationship between cholesterol concentration and the membrane activity of BAPs.

When looking at cholesterol–peptide interactions, a number of questions come to mind. Do BAPs interact specifically with cholesterol leading to inhibition of the insertion process? In this case, the inhibition would be specific to a group of peptides. Does cholesterol induce inhibition of peptide activity indirectly through shifts in the mechanical properties of the membrane? This would lead to a broad inhibition of peptide activity independent of the BAP mechanism of insertion. Table 2 summarizes some research works that studied the effect of cholesterol on the activity of membrane-active peptides. The pore-forming activity of several of the alpha-helical BAPs presented in Table 2, such as melittin and magainin, is inhibited in the presence of cholesterol. However, this inhibitory effect is also seen in Protegrin-1, which forms a beta hairpin when interacting with the bilayer. Gramicidin also shows inhibition. This peptide, in particular, requires dimerization in the membrane of two subunits to construct a membrane spanning beta helix, which acts as an ion selective channel. Dimerization normally requires local deformation of the membrane due to a hydrophobic mismatch between the thickness of the membrane and the length of the gramicidin dimer. Since cholesterol rigidifies the membrane, this would result in a greater energy cost to induce the deformation in order to account for the mismatch. The main message is that, due to the regulation of the mechanical properties of membranes by cholesterol, BAP activity, which requires membrane deformation, in general will be inhibited by the presence of this lipid molecule.

The activity of BAPs appears to be closely connected to the mechanical properties of the membrane, and a more resilient membrane, presenting a higher bending rigidity, will increase the cost of deformation and therefore inhibit BAP activity. Due to the connection between the energy cost associated with deformation and the chemical activity of the BAPs, a membrane with increased rigidity will require higher concentrations of BAPs in the media to achieve insertion. In addition to increasing the acyl chain order, it has been established that cholesterol can remove the tilt angle of the hydrophobic tails in the lipid bilayer, which will also induce membrane thickening, leading to inhibition of BAP activity. With a face-hinged cyclohexane ring, cholesterol can reduce the van der Waals attractive energy between adjacent lipid tails. However, cholesterol will induce its own interactions with neighboring lipids, and, since cholesterol is rigid, the lipid acyl chains will have to pay a higher energy price to bend away from cholesterol in the process of inducing membrane curvature. This would be considered an additional energy cost to bending, and would further inhibit the activity of BAPs [125].

The protective effect of cholesterol is peptide dependent. In a recent study using two synthetic peptides, it was shown that the presence of high cholesterol levels, such as those found in red blood cells, prevented peptide-induced thinning as measured by X-ray diffraction. In the same study, bacterial cell models were used and compared with minimum inhibitory concentrations in Gram-positive and Gram-negative bacteria, showing that thinning was correlated with toxicity. However, for the cholesterol containing membranes, even though thinning was prevented in the presence of cholesterol, only one of the two peptides showed strongly reduced hemolytic activity, which appeared to indicate that toxicity is not necessarily correlated to BAP-induced membrane thinning [126]. On the other hand, peptide localization within the membrane was measured through neutron diffraction, where cholesterol appeared to inhibit peptide penetration into the membrane’s core region. In addition, the secondary structure of the peptides showed significant changes in the red blood cell model, presenting reduced formation of alpha helical structures. The study was complemented by NMR spectroscopy showing changes in headgroup mobility, and differential scanning calorimetry showing downward shifts in the melting temperature of the membrane in the presence of the peptides [127].

These structural studies provide very valuable information with regards to peptide positioning and changes in membrane structural parameters, comparing these with minimum inhibitory concentrations and toxicity studies on live cells, in particular when comparing bacterial and eukaryotic models. However, certain structural details cannot be teased out in full detail with these techniques. A new approach is to use scattering guided molecular dynamic simulations to be able to gain more insight into the response of the membrane in the presence of these antimicrobial peptides [127]. While molecular dynamics provides exquisite molecular detail, it falls short in some cases in accurately representing the physical parameters of the membrane. By using X-ray diffraction to stir the molecular dynamic simulations, the physical parameters can be represented more closely, which leads to a better representation of the interaction of the peptides with membrane presenting different compositions.

Charge has always been thought of as the main discriminating factor in the selectivity of antimicrobial peptides towards bacterial membranes. However, hydrophobic interactions can play an important role in peptide binding to the membrane. For this reason, it is important to consider other structural factors to induce preferential binding towards bacterial membranes versus eukaryotic membranes. The exclusive presence of cholesterol in eukaryotic membranes can provide an additional parameter to induce selectivity. However, a better understanding of how cholesterol influences BAP lytic activity is necessary to have a clear picture when designing antimicrobial peptides. This requires multiple experimental techniques to elucidate changes in the structural parameters of the membrane associated with peptide adhesion and insertion, and location of the peptide within the membrane. These experimental results should be complemented with molecular dynamics to obtain a more detailed structural picture of this interaction. These structural studies should be correlated with leakage experiments in liposomes made of model systems and cell lipid extracts and compared with minimum inhibitory concentrations in bacteria and toxicity measurements in eukaryotic cells. Cholesterol is a promising candidate to push towards a higher level of selectivity for antimicrobial peptides. The structural role that cholesterol plays in interfering with peptide activity needs to be studied in more detail to fully understand its potential in increasing peptide selectivity.

## 6. Conclusions

Despite the unquestionable importance of cholesterol, very few studies on the interaction between BAPs and cholesterol have been published as yet, but such interaction is important; as approximately 30% of the steroids in the mammalian plasma membrane consist of cholesterol, it is likely that a significant proportion of BAPs encounter this molecule (in addition to frequently meeting a sphingolipid component). In summary, the interaction of AMPs with previously proposed plasma membrane microdomain components (sphingolipids, glycolipids, cholesterol) is most relevant when designing experiments with a view to mimicking native membrane properties. Interactive work concerning the interaction between AMPs and cholesterol may also lead to novel insights.

## Figures and Tables

**Table 1:** Summary of studies reporting the quantification of lipids of the outer layer (OL) of the membrane of eukaryotic cells.

Cell Type	Results OL	Unit	Technique Used for Quantification	Year	Ref.
Erythrocytes	PC: 8.5 SM: 9.3 PS: 0.48	Mol %	Enzymatic digestion/Mass spectrometry	2023	[65]
	PC: 1 SM: 1	Ratio	Cyclodextrin chemistry/Thin-layer chromatography/Mass spectrometry	2020	[66]
	PC: 42 SM: 48 PE: 1 PS: 3	Mol %	Enzymatic digestion/Mass spectrometry	2020	[67]
	PC: 98 SM:99	Total PL (%)	Sodium dodecyl sulphate-digested freeze–fracture/Specifics monoclonal antibodies	2018	[68]
	PC: 98 SM: 95	Total PL (%)	Sodium dodecyl sulphate-digested freeze-fracture/Specifics monoclonal antibodies	2015	[69]
	PC: 19.2 SM: 20 PE: 7 PS: 1.3	Total PL (%)	Not reported	2012	[70]
	PC: 22.5 SM: 21.1 PE: 7	Total PL (%)	Not reported	2008	[71]
	PC: 15.1 SM: 27.7	Total PL (%)	Not reported	2000	[72]
	PC: 61.5 SM: 85 PE: 4.5 PS: 0	Total PL (%)	Enzymatic degradation/Thin-layer chromatography and determined as phosphorus	1975	[73]
	PC: 21 SM: 20.5 PE: 5.7	Total PL (%)	Enzymatic degradation and freeze-etching electron microscopy/Two-dimensional thin-layer chromatography and determined as phosphorus	1973	[74]
	PE: 33 PS: 0	Total PL (%)	Titrobenzenesulfonate-diazosulfanilic acid/UV–Vis spectrometry	1973	[75]
	PC: 3 SM: 2	Ratio	Enzymatic degradation/Two-dimensional thin-layer chromatography and UV–Vis spectrometry	1973	[76]
Platelets	PC: 6.5 SM: 11.7 PE: 1.8 PS: 0.3	Total PL (%)	Enzymatic degradation	1987	[77]
	PC: 45.9 SM: 41.8 PE: 10.8 PS: 0.5	Total PL (%)	Enzymatic degradation/Thin-layer chromatography and determined as phosphorus	1986	[78]
	PC: 31 SM: 57.1 PE: 9.5 PS: 2.4	Total PL (%)	Enzymatic degradation/Thin-layer chromatography and determined as phosphorus	1983	[79]
	PC: 12.7 SM: 23.8 PE: 9.4 PS: 1.4	Total PL (%)	Enzymatic degradation/Thin-layer chromatography and determined as phosphorus	1977	[80]
	PC: 26.7 SM: 4.2 PE: 17.3 PS: 2.8	Total PL (%)	Enzymatic degradation/Thin-layer chromatography and determined as phosphorus	1976	[81]
	PE: 17.9 PS: 0	Total PL (%)	Titrobenzenesulfonate/Thin-layer chromatography and determined as phosphorus		[82]
Others	PC: 60.1 SM: 3.3 PE: 11.9 PS: 9.9	Total PL (%)	Cyclodextrin chemistry/Thin-layer chromatography/Mass spectrometry	2016	[83]
	PC: 82.3 SM: 87.8 PE: 2.5 PS: 4.2	Total PL (%)	Sodium dodecyl sulphate-digested freeze-fracture/Specific monoclonal antibodies	2015	[69]

**Table 2:** Summary of research works that analyze the potential modulatory effect of cholesterol on the activity of bioactive peptides. Ornithine (O*), D-Phenylalanine (F*).

Peptide	Sequence	Net Charge	Technique	Result	Ref.
Melittin	GIGAVLKVLTTGLPALISWIKRKRQQ	+6	Dynamic giant uni-lamellar vesicle leakage assay and coarse-grained (CG)/Fluorescence spectroscopy/Molecular dynamics (MD)	A higher peptide concentration is necessary to induce calcein release in the DOPC/CHO system as opposed to the membrane model with DOPC alone. CHO hinders peptide-induced pore formation in the membrane model	[115]
Protegrin-1 (PG-1)	RGGRLCYCRRRFCVCVGR-NH_2_	+7	Isothermal titration calorimetry (ITC) and atomic force microscopy (AFM)	Increasing CHO content decreases the favorability of the peptide–lipid interaction	[116]
Amyloid-beta (Aβ)	DAEFRHDSGYEVHHQKLVFFAEDVGSNKGAIIGLMVGGVVIA	−3	MβCD-cholesterol complex/Cell viability assay (MTT)/Patch-perforated/Generalized polarization (GP)	Effect on Aβ depends on the amount of CHO in the membranes. Low CHO induced a facilitation of the membrane perforation andhigh CHO inhibited membrane disruption	[117]
Melittin	GIGAVLKVLTTGLPALISWIKRKRQQ	+6	Small-angle neutron scattering (SANS)/Circular dichroism (CD)	Increasing CHO content decreases the amount of transmembrane peptide. CHO reduces the penetration depth of melittin, which would decrease the amount by which it thins the bilayer	[118]
Melittin	GIGAVLKVLTTGLPALISWIKRKRQQ	+6	Circular dichroism (CD)/Elastic incoherent neutron scattering (EINS)/Quasi-elastic neutron scattering (QENS)	Adding CHO to DMPC vesicles mitigates peptide interaction and prevents melittin embedding in the membrane depth	[119]
MSI-78 MSI-594 MSI-367 MSI-843	GIGKFLKKAKKFGKAFVKILKK-NH_2_ GIGKFLKKAKKGIGAVLKVLTTGL-NH_2_ KFAKKFAKFAKKFAKFAKKFA-NH_2_ Oct-OOLLOOLOOL-NH_2_	+10 +7 +10 +7	Fluorescence spectroscopy	A strong reduction in membrane alteration is only observed for all peptides tested above 20% CHO content. CHO’s protective, membrane stabilizing effect does not occur to an appreciable level in lipid systems containing raft domains	[120]
Cys-TP	GWTLNSAGYLLGCINLKALAALAKISIL-NH_2_	+3	Spin-label electron paramagnetic resonance (EPR)	An increase in CHO in the DMPC lipid system resists the incorporation of Cys-TP into the lipid bilayer	[121]
DD K	H-GLWSKIKAAGKEAAKAAGKAALNAVSEAV-NH_2_	+5	Surface plasmon resonance (SPR)/Atomic force microscopy (AFM)/Isothermal titration microcalorimetry (ITC)/Fluorescence spectroscopy/Dynamic light scattering (DLS)	Peptide interaction with mimetic PC membranes is reduced by CHO presence in concentrations typically found in mammalian cells	[122]
Magainin 2 Indolicidin	GIGKFLHSAKKFGKAFVGEIMNS ILPWKWPWWPWRR	+4 +3	Magnetically stirred circular Teflon wells/Fluorescence spectroscopy	The CHO presence prevents the membrane-perturbing action of magainin 2 and, in the same system with CHO, indolicidin exerts a disturbing effect on the membrane model	[123]
Gramicidin S	Cyclo-(VO*LF*P)_2_	-	Fourier transform infrared spectroscopic (FT-IR)/Circular dichroism (CD)/Nuclear magnetic resonance (^31^ P-NMR)/Fluorescence spectroscopy	The presence of CHO attenuates the interaction of GS with PC bilayers. CHO-containing POPC vesicles are more resistant to peptide-induced permeabilization than CHO-free vesicles	[124]

## Data Availability

The data involved in this paper are presented in articles and supporting materials in the form of diagrams or tables.
